# Parent–adolescent communication quality does not moderate the association of emotional burden and somatic complaints in adolescents: a cross-sectional structural equation model

**DOI:** 10.1186/s13034-025-00882-9

**Published:** 2025-03-22

**Authors:** Holger Zapf

**Affiliations:** Department of Child and Adolescent Psychiatry, Psychosomatics and Psychotherapy, University Medical Center Hamburg-Eppendorf, 20246 Hamburg, Germany

**Keywords:** Parent–adolescent communication, Mental health, Somatoform disorder, Parent–adolescent relationship, Internalizing disorder

## Abstract

An explanation for somatic complaints in adolescence assumes that they have the function to express emotional burden if the communication of feelings in important relationships does not work sufficiently. Therefore, it can be hypothesized that in adolescents, lower quality of emotional communication with a parent goes along with a higher impact of emotional burden on somatic complaints. The aim of this study was to examine whether emotional communication quality between adolescents and parents moderates the association of emotional burden and somatic complaints. Based on data from a cross-sectional population sample (N = 1061), structural equation modeling (SEM) was used to test the hypothesis. In addition to the general model, models for boys and girls were compared. Emotional communication quality does not moderate the association of emotional burden and somatic complaints in the general model or in the gender-based models. However, communication quality is a significant predictor of somatic complaints for boys. Limitations are the cross-sectional nature of the data, the possible sampling bias due to the use of an online access panel, and the inclusion of one parent per adolescent. This study highlights that emotional communication quality is a predictor for somatic complaints in adolescent boys and should be addressed in therapy.

*Trial registration* ClinicalTrials.gov: NCT05332236.

## Highlights

- A structural equation model was conducted on a data from a population sample.
- Emotional burden predicts somatic complaints across gender groups in adolescents.
- Parent–adolescent communication quality does not moderate this association.
- Communication quality predicts somatic complaints in boys, but not girls.

## Introduction

Somatic complaints in adolescence are common [1, 2]. Medically unexplained complaints like headaches or stomachaches can lead to considerable distress for those affected and this, in turn, can cause overutilization of health care [3] and substantial costs in the health care system [4]. In addition, chronic somatic complaints in adolescence predict severe mental illness in adulthood [5]. Therefore, it is important to understand the mechanisms that lead to somatic complaints and improve care [6].

In mental health frameworks, medically unexplained somatic complaints are usually interpreted as somatoform symptoms or somatization [7, 8]. Somatoform symptoms are generally considered to be related to psychological distress [9]. In the context of clinical child and adolescent psychology, they are classified as internalizing disorders alongside depression or anxiety, and comorbidites with other internalizing disorders are common [10–12].

Depending on the respective therapeutic school, different hypotheses of causal factors have been specified. Among others, learning effects, prolonged attention to bodily sensations, alexithymia, disorders of emotion regulation and attachment as well as problems in the family system have been discussed [13–18]. Also, several risk factors for somatic complaints have been identified in the past, including socio-economic status [19], illness in early childhood and anxious parental illness behavior [14], somatic anxiety [12], and family risk factors such as parents with a somatoform disorder, close relatives with a severe illness [20, 21], or poor family functioning [22].

Several models have been formulated to explain the etiology of somatoform symptoms [17, 23]. One explanatory model that is partly in line with other models [24, 25] and is operable across different therapeutic schools is based on the assumption that there is a vicious cycle between low parental responsiveness (or, in other words, low sensitivity to attachment needs) and emotional distress of a child. If emotional distress is not met by an adequate parental response, somatic symptoms that accompany emotional distress may be presented and elicit parental attention. Once the child learns that this pathway leads to attention, this behavior is likely to be reinforced, just as the parents learn that they can satisfy their child’s needs by reacting to somatic complaints [26]. In this sense, somatic complaints can be considered to have a communicative function [27], indicating that the sender needs attention and is not capable to communicate emotional needs sufficiently otherwise—together with a potential side effect of focusing away from areas of interpersonal conflict [14, 16].

The assumption that somatic complaints may have a communicative function in parent–adolescent relationships has, to our knowledge, not yet been tested empirically. However, it is supported by the related finding that psychotherapeutic interventions focusing on the improvement of parent–adolescent communication quality effectively reduce somatoform symptoms [28]. In addition, research on children aged seven to eleven years also found that somatic complaints increase with decreasing communication quality [29], and a meta-analysis found a small but significant effect of attachment on somatoform symptoms for both children and adolescents [30].

### Hypotheses

The current exploratory study aims to investigate the possible communicative function of somatic symptoms in a population sample using a structural equation model (SEM) based on cross-sectional data. The model tests the following hypotheses: (1) Emotional distress predicts somatic symptoms in adolescents [31]. (2) If somatic symptoms increase because interpersonal communication of emotional distress is not possible in a sufficient way, parent–adolescent communication quality moderates this association in parent–adolescent dyads. Hence, lower quality of dyadic emotion communication quality should lead to a higher impact of emotional distress on somatic symptoms. (3) Parental somatic symptoms increase the likelihood of adolescent somatic symptoms [32]. Since boys and girls have shown different patterns of somatization and psychosocial predictors in previous studies [10, 33], gender-based SEMs will be calculated in addition to the general model.

## Method

### Participants and procedure

This study is based on a population sample that was recruited in Germany through an online access panel with the assistance of a demographic consulting company for market and social research (Bilendi). Data were collected between September and October 2022. Registered adults with children aged ten to 18 years were asked to participate. They were screened for parent gender, parental education, and adolescent gender. Quotas were set for the screening questions in order to achieve a distribution similar to the German population [34]. The survey consisted of two parts: First, the parent or caregiver filled in questionnaires, then they were asked to leave the online device to the child to complete the questionnaires for adolescents on their own. Each parent could only take part with one child. If parents had more than one eligible child, they were asked to choose the child that they had thought about most often recently. After the completion of the survey, participants received an incentive. The sample consisted of 1061 parent–child dyads with 58.2% female caregivers, age M (SD) = 43.3 (7.7) years; and 49.2% female adolescents, age M (SD) = 13.4 (2.2) years (Table 1).

**Table 1:** Sociodemographic characteristics

	Parent* N* = 1061 Adolescent* N* = 1061	
Parents/caregivers	*M*	*SD*
Age (years)	43.3	7.7
Female (n = 600)	42.4	6.9
Male (n = 459)	44.6	8.4
Non-binary (n = 2)	31.0	8.5
Number of children in family	1.9	1.2

	*n*	%
Mothers	617	58.2
Step-/foster mothers	21	2.0
Fathers	442	41.7
Step-/foster fathers	42	4.0
Marital status		
Married	713	67.2
Living with a partner	128	12.1
Single	120	11.3
Divorced	86	8.1
Widowed	14	1.3
Educational status (mothers/fathers)		
Higher education qualification	418	39.4
General certificate of secondary education	375	35.3
Secondary education	238	22.4
Other	13	1.2
None	16	1.5
Not stated	1	0.1
Professional qualification (mothers/fathers)		
Vocational training	603	56.8
Master handicraft	145	13.7
University	169	15.9
Other	48	4.5
None	96	9.0
Single parent	221	20.8
Other language than German at home	42	4.0

Adolescents	*M*	*SD*
Age (years)	13.4	2.2
Female (n = 522)	13.4	2.2
Male (n = 529)	13.4	2.2
Non-binary (n = 10)	15.2	2.6

Ethics approval was obtained from the Local Ethics Committee of the Center for Psychosocial Medicine, Hamburg-Eppendorf Medical University Center (LPEK-0396). All parents and adolescents provided informed consent. Participants could withdraw from the study at any time. The current analysis is a prespecified secondary analysis of data from a psychometric study that was preregistered at ClinicalTrials.gov (NCT05332236). Therefore, it is an exploratory analysis.

### Measures

The quality of dyadic parent–adolescent emotional communication was assessed with items from the German translation of the Parent–Adolescent Communication Scale (PACS) [35, 36]. The original adolescent self-report measure consists of 20 identical items that are rated on a scale from 1 (“strongly disagree”) to 5 (“strongly agree”). Higher values indicate higher communication quality. The items show acceptable to excellent internal consistencies across many studies [37]. Scalar measurement invariance was established across gender groups [36]. To represent the latent construct of dyadic emotional communication quality, seven items of the original scale were identified according to item content and narrowed down to three items according to an analysis of covariance patterns and standardized residuals. These items were “My mother/father is always a good listener”, “I am very satisfied with how my mother/father and I talk together” and “It is very easy for me to express all my true feelings to my mother/father”.

Items from the German version of the Strengths and Difficulties Questionnaire [38, 39] were used to assess emotional burden (adolescent self-report). The measure consists of 25 items that are rated from 0 (not true) to 2 (certainly true). Four problem subscales can be calculated, with higher values indicating higher burden. Four items from the emotional symptoms subscale were considered to represent emotional burden in the SEM. According to an analysis of covariance patterns and standardized residuals, three items were chosen to represent the latent construct.

The Somatic Complaints Subscale (SCS) from the German version of the Youth Self-Report (YSR) [40] was used to assess somatic symptoms (adolescent self-report). It consists of seven items and is rated from 0 (not true) to 2 (very true or often true). The results of each item were added up to assess somatic symptom severity. The Somatic Complaints Subscale has already been used in previous studies and shows acceptable internal consistency [41].

The physical health component scale of the German version of the Short Form SF-12 [42] was used to assess the physical quality of life of the parent (parent self-report). Higher values indicate better quality of life. Since the items of the SF-12 are measured on different scale levels, the computed score was used as a single variable in the structural equation model. The physical health component scale shows good psychometric properties and the underlying measurement model and dimensional structure has been confirmed [43]. Socio-demographic variables included the parents’ gender, age, family situation and education, which were self-reported by the parents. Gender was measured on a three-step scale (female, male, non-binary), family situation was assessed with the items number of children in the household and parental relationship status. Socioeconomic status was assessed in terms of parental school education and job training. Adolescents were asked to self-report their age and gender.

### Data preparation

Complete straightliner (variance = 0 in raw items of questionnaire in parent or adolescent section respectively) and speeder cases (time to complete < 520 s in the population sample) were deleted. Reports on referenced attachment figure (mother/father/stepmother/stepfather/foster mother/foster father/other) as well as gender of the attachment figure and age of the child compared to age of attachment figure were checked for plausibility, and inconsistent cases were deleted. Since item completion was mandatory, missing data only occurs due to straightlining and is rare (< 1.5%).

### Statistical analysis

The hypothesis of a moderator effect between emotional burden and somatic complaints was tested in the framework of a structural equation model (SEM). In a preliminary step, items representing the latent constructs were identified and covariance and correlation patterns as well as residual variances of the path model were inspected [44]. Items that did not load sufficiently on the latent constructs or contributed to high residual variances were excluded from the analysis. In addition, multicollinearity of the resulting indicators was tested, with variance inflaciont factor (VIF) values > 5 indicating critical multicollinearity. Following the two-step approach of Anderson and Gerbing [45], a confirmatory factor analysis (CFA) for the general model was conducted to assess model fit. Since the χ^2^ test statistic tends to reject model fit in large samples [46], the model fit was evaluted on the basis of the following indices: Comparative Fit Index (CFI), Tucker-Lewis Index (TLI), Root Mean Square Error of Approximation (RMSEA), and Standardized Root Mean Square Residual (SRMR) [47]. The χ^2^ test statistic was reported additionally. Thresholds for good model fit were > 0.95 for CFI and TLI (> 0.90 is acceptable), RMSEA < 0.06 (< 0.09 is acceptable), and SRMR < 0.08.

In the second step, the general SEM was specified and tested. Since the maximum likelihood estimator is considered to be robust against violations of normality assumptions and does not overestimate model fit in contrast to the diagonally weighted least squares estimator [48], estimates were obtained with the maximum likelihood function with robust standard errors and Yuan-Bentler correction [49]. Missing data was estimated with full information maximum likelihood. As suggested by Steinmetz [44], the interaction term was estimated based on the covariances of all items indicating the primary factors (emotional burden and dyadic emotional communication quality) and covariances between the primary factors and the interaction term were set to zero. Measurement error terms for latent constructs indicated by single values (parental physical quality of life and adolescent somatic complaints) were set to 10% of the variance of the respective variables.

Since previous research found differences in the patterns of somatic symptoms and predictors of male and female adolescents [33], gender groups were analyzed seperately in addition to the general SEM. Analyses were run in R, version 4.3.2. The SEM was conducted with the lavaan package, version 0.6.17.

## Results

Preliminary Analyses. Since item 13 of the SDQ and items 4, 8, 11, and 16 of the PACS showed high residual variances and/or low factor loadings, they were not included in the SEM. Parental educational status was not associated to the other variables and was also not included in the analysis. For the remaining items and the respective summary scales, no signs of multicollinearity were found (see Table 2).

**Table 2:** Preliminary analyses: Variance inflation factors (VIF)

Variable	Reported by	VIF	1	2	3
1. Emotional Communication	Adolescent	1.08			
2. Emotional burden	Adolescent	1.11	− 0.26		
3. Parental somatic quality of life	Parent	1.05	0.11	− 0.22	
4. Somatic complaints	Adolescent	–	− 0.25	0.47	− 0.25

Confirmatory Factor Analysis (CFA). The general model showed sufficient fit properties without the interaction term. As expected, the χ^2^ test statistic (49.7, df = 16) was significant and rejected model fit. However, all other fit measures indicated good to excellent model fit: CFI = 0.98, TLI = 0.96, RMSEA = 0.05, SRMR = 0.02. All factor loadings showed sufficient standardized loadings > 0.4.

**Fig. 1:**
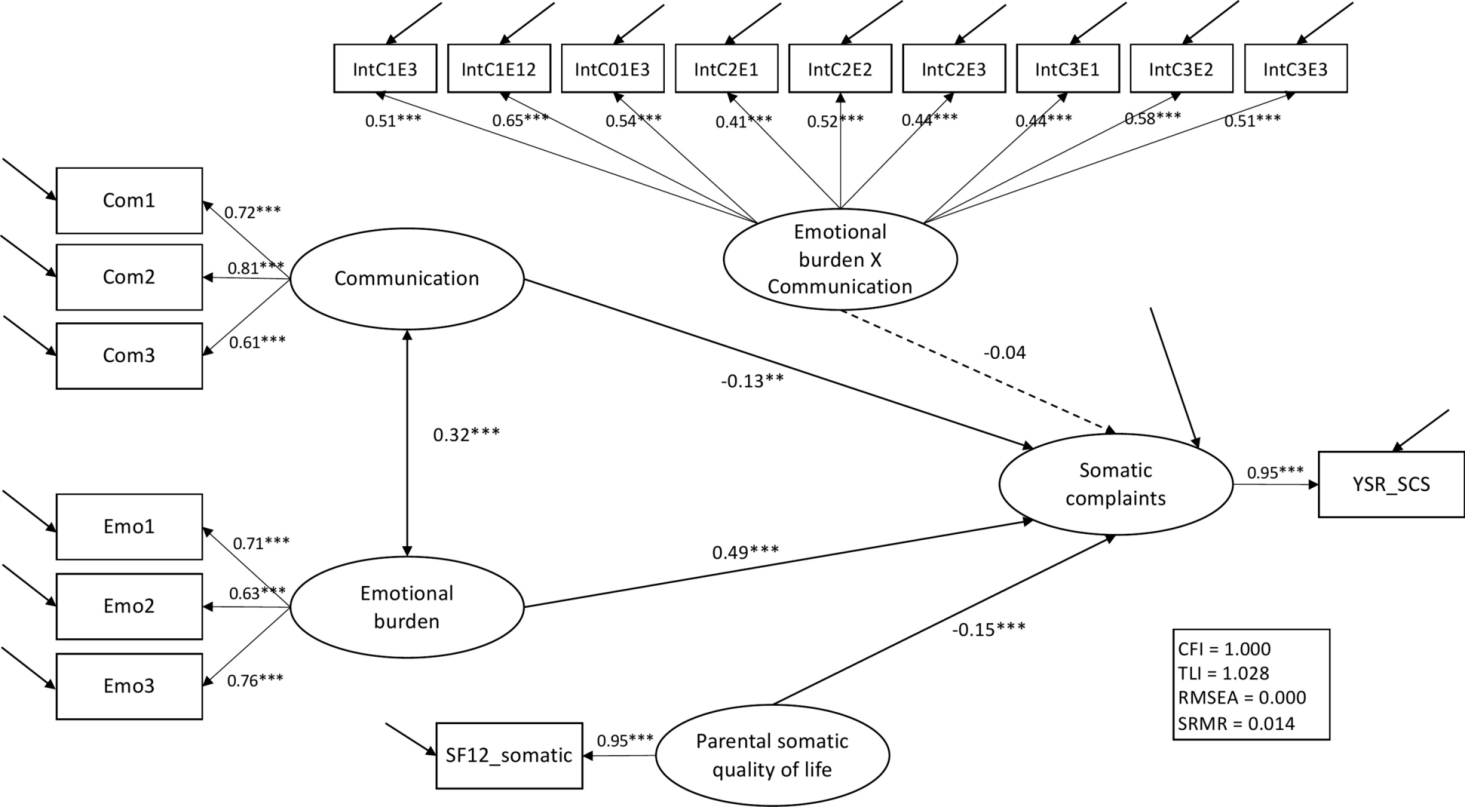
General structural equation model on the moderating effect of dyadic emotional communication quality on the association of emotional burden and somatic complaints. Correlations outside the hypothetical model have been omitted for the sake of clarity. *p < 0.05, **p <.0.01, ***p <.0.001

General SEM. The general SEM included all latent constructs (see Fig. 1). Since the degrees of freedom exceeded the χ^2^ test statistic (37.8, df = 95), none of the fit measures indicated model misfit (*p*_χ2_ = 1.0, CFI = 1.0, TLI = 1.0, RMSEA < 0.01, SRMR = 0.01). Adolescent emotional burden (β = 0.49, *p* < 0.001), adolescent-reported dyadic emotional communication quality (β = − 0.13, *p* = 0.001), and parental somatic quality of life (β = − 0.15, *p* < 0.001) were all relevant predictors of adolescent-reported somatic symptoms. The interaction term of emotional burden and emotional communication quality was not a significant predictor of somatic symptoms (β = − 0.04, *p* = 0.36).

SEM with gender groups. The gender group SEM showed good model fit (χ^2^ test statistic = 284.4, df = 306, *p*_χ2_ = 0.81, CFI = 1.0, TLI = 1.0, RMSEA < 0.01, SRMR = 0.05). Scalar measurement invariance between the groups could be established (configural model vs. metric model: *p*_χ2_ = 0.31, Δχ^2^ = 13.9, Δdf = 12; metric model vs. scalar model: *p*_χ2_ = 0.36, Δχ^2^ = 4.3, Δdf = 4). In the group of adolescent girls, adolescent emotional burden (β = 0.46, *p* < 0.001) and parent somatic quality of life (β = − 0.15, *p* = 0.003) were relevant predictors of adolescent-reported somatic symptoms. Adolescent-reported dyadic emotional communication quality (β = − 0.06, *p* = 0.28) and the interaction term of emotional burden and emotional communication quality were not significant predictors of somatic symptoms (β < − 0.01, *p* = 0.93). In the group of adolescent boys, adolescent emotional burden (β = 0.50, *p* < 0.001), adolescent-reported dyadic emotional communication quality (β = − 0.18, *p* = 0.001) and parent somatic quality of life (β = − 0.15, *p* = 0.001) were relevant predictors of adolescent-reported somatic symptoms. The interaction term of emotional burden and emotional communication quality was not a significant predictor of somatic symptoms (β < − 0.03, *p* = 0.64).

## Discussion

In previous research, somatic complaints were sometimes considered to have a communicative function, indicating that the sender is not capable to communicate emotional needs sufficiently otherwise [14, 16, 26]. The purpose of this paper was to investigate this possible communicative function of somatic symptoms with cross-sectional data from a population sample using structural equation modeling (SEM). (1) It was hypothesized that emotional burden predicts somatic symptoms, this hypothesis cannot be rejected based on the model. Emotional burden was a major predictor of somatic symptoms as expected from previous research [11, 12]. (2) It was hypothesized that the interaction of emotional burden and communication quality moderates the association between emotional burden and somatic symptoms. No evidence for this hypothesis was found in the analysis of the data. (3) Parental somatic quality of life also predicted somatic symptoms in children to a lesser extent. This is also in accordance with previous findings [20].

To our knowledge, this study is the first to assess the association between somatic symptoms and parent–adolescent communication quality [50]. In the general SEM, the effect size of dyadic emotional communication quality on somatic symptoms was comparable to that of parental somatic quality of life. Thus, the quality of parent–adolescent communication is probably not irrelevant for somatic symptoms even if there is no moderation effect. However, the association may be more trivial than originally assumed. In the current sample, no evidence for the moderation hypothesis was found, indicating that worse communication quality does not increase the impact of emotional burden on somatic symptoms in a significant way. In other words, the current SEM gives no hint that somatic symptoms have a communicative function to articulate emotional distress in contexts of difficult dyadic emotional communication. Rather, lower dyadic emotional communication quality may simply be a stressor itself that contributes to higher somatic symptom load [29].

When the paths of the gender group SEMs are inspected, different patterns between adolescent boys and girls can be identified. This is in accordance with previous research as patterns of predictors seem to differ generally [51]. It is also not unusual to find different psychosocial effects of parent–adolescent communication quality on adolescent boys and girls [52, 53]. In the current analysis, the SEM for girls did not show an impact of dyadic emotional communication quality on somatic symptoms, whereas its effect exceeded that of parental somatic quality of life in the SEM for boys.

According to the nature of the data and the analysis, this finding should not be overintepreted. A possible explanation may be that adolescent girls have more interpersonal ressources to compensate family distress that is caused by dyadic communication difficulties with parents, as their friendships are more intimate and validating [54]. However, a recent study shows that parents are more important than peers for interpersonal emotion regulation in adolescent girls [55]. The difference may also be explained with regard to particularities of male adolescents. Adolescent boys may be expected not to express emotional problems toward their parents as part of their social gender role [56], leaving somatic complaints as a more suitable valve to react to distress that results from low dyadic emotional communication quality. Since the data are cross-sectional, a third explanation is that girls have better chances to elicit improvements in parent–adolescent communication than boys when they show somatic symptoms. Either way, the impact of communication difficulties on boys’ somatic complaints is consistent with the finding that a psychotherapeutic focus on parent–adolescent communication quality is helpful to reduce somatoform symptoms [28]. Further studies should consider gender differences with regard to this effect.

## Limitations

The most important limitation of the current study is the cross-sectional nature of the data: No causal conclusions can be drawn from the analysis. Further studies should utilize longitudinal data with the independent variables clearly preceding the dependent variable in time. This would also allow to specify the structural model differently, since emotional communication quality itself may as well have an influence on emotional burden in adolescents [50] and should therefore be measured at baseline and as an intermediate variable together with emotional burden. Another major limitation is that potential confounders such as major life events, family cohesion or general socioeconomic status were not considered in the model. Also, emotional communication quality was measurend only with one parent/caregiver instead of both. The second parent can compensate for problems in the other dyad. Furthermore, the secondary analysis focused on a comparison of models based on adolescent gender and established measurement invariance, but parent and adolescent gender was not accounted for in the general model. Therefore, the results do not allow conclusions about gender interaction and should thus not be overinterpreted. The sample was drawn from the general German population. It is possible that the pathway between emotional distress, communication and somatic complaints shows different properties in clinical populations or families with low functioning. This is relevant especially since these are the target populations of psychotherapeutic interventions and consulting. Last but not least, the sample was recruited via an online access panel. Therefore, the sample is not random as sampling bias may have occured during the initial acquisition of the panel.

## Conclusion

Parent–adolescent emotional communication quality appears not to be a moderator of the association between emotional burden and somatic complaints. However, emotional communication quality is a factor to be considered in research and treatment of adolescent somatic complaints. Especially in psychotherapy with male adolescents, an additional focus on parent–adolescent communication may be helpful to reduce somatic complaints. However, intervention studies would be necessary to substantiate this assumption. Future studies should investigate clinical samples or population samples in other countries, gather longitudinal data, and be sensitive to differences regarding adolescent gender.

## Data Availability

The datasets generated during the current study are available from the corresponding author upon reasonable request.

## Abbreviations

SEM: Structural equation modeling
VIF: Variance inflation factor
